# Prognostic relevance of CD163^+^ immune cells in patients with metastatic breast cancer

**DOI:** 10.1007/s00262-024-03892-2

**Published:** 2025-01-03

**Authors:** Ida Lindberg, Aya Saleh, Julia Tutzauer, Frida Björk Gunnarsdottir, Lisa Rydén, Caroline Bergenfelz, Anna-Maria Larsson

**Affiliations:** 1 Department of Translational Medicine, Division of Experimental Infection Medicine, Lund University, 221 84 Lund, Sweden; 2 Department of Clinical Sciences Lund, Division of Oncology, Lund University, 221 84 Lund, Sweden; 3 Department of Oncology and Pathology, Karolinska Institute and University Hospital, Stockholm, Sweden; 4 Breast Center, Karolinska Comprehensive Cancer Center, Karolinska University Hospital, Stockholm, Sweden

**Keywords:** Metastatic breast cancer, Prognosis, CD163, Myeloid cells, Tumor-associated macrophages

## Abstract

Metastatic breast cancer (MBC) is generally considered an incurable disease and even though new treatments are available, the median survival is approximately three years. The introduction of immune therapies for MBC highlights the importance of the immune system in cancer progression and treatment. CD163^+^ anti-inflammatory myeloid cells, including tumor associated macrophages (TAMs), are known to be of relevance in early breast cancer but their role in MBC is not yet established. Here we determine the levels of CD163^+^ immune cells in 139 patients with newly diagnosed MBC by using Immunohistochemistry (IHC) and gene expression analyses (GEX). We aim to determine changes and distribution of CD163^+^ immune cells during tumor progression from primary tumors (PT) to lymph node metastases (LNM) and distant metastases (DM). In addition, we evaluate associations between CD163^+^ immune cells, clinicopathological factors and disease outcome (progression-free and overall survival; PFS and OS, respectively). Despite similar distribution, high levels of CD163^+^ immune cells in the tumor nest of PT, but not in LNM or DM, associated with adverse prognostic features including higher grade and molecular subtype, as well as with shorter PFS and OS, however this observation was not significant after adjusted multivariate analyses. Finally, high levels of CD163^+^ immune cells in PT, as well as GEX in PT and synchronous LNM associated with shorter OS from the initial diagnosis. These results indicate that evaluating the levels of CD163^+^ immune cells may identify MBC patients with a worse prognosis. Unraveling the role of CD163^+^ immune cells in the complex immune responses in MBC is highly relevant for improving future immune therapies.

**Supplementary Information:**

The online version contains supplementary material available at 10.1007/s00262-024-03892-2.

## Introduction

Although advances in diagnostics and treatments have improved the outcome of patients with primary breast cancer, approximately one in three will still develop metastatic disease [1]. Metastatic breast cancer (MBC) is generally considered incurable with an estimated median overall survival (OS) of approximately two to three years, and a five-year survival rate of merely 25% [1, 2].

Predicting prognosis and treatment response in MBC is clinically challenging, highlighting a need for novel biomarkers to improve tailoring of individualized therapies [1, 2]. The immunological composition of the primary tumor microenvironment has gained attention, with distinct cellular populations conferring prognostic information in primary breast cancer [3]. However, the dynamics of the immune landscape during tumor progression and its clinical relevance in metastatic tissue remains unclear. Investigation of the immune landscape in both lymph node and distant metastases is crucial for understanding tumor progression in MBC. In addition, with the rapid development of immune therapies, insights into the complex interactions between cancer cells and immune responses in MBC is urgently needed.

CD163 is a scavenger receptor expressed on anti-inflammatory cells of the myeloid lineage. While being a common marker for tumor associated macrophages (TAMs), CD163 may also be expressed on monocytic myeloid-derived suppressor cells (Mo-MDSCs) [4, 5]. Numerous studies have indicated that presence of CD163^+^ immune cells is associated with clinicopathological features and prognosis in a wide range of malignancies, including breast cancer [4, 6, 7]. In patients with primary breast cancer, significant associations have been made for high densities of CD163^+^ immune cells and higher tumor grade, larger tumor size, lymph node positivity, Ki67-positivity, hormone receptor negativity and/or triple negative/basal like subtypes [7–12].

In accordance with the associations to adverse clinicopathological features, high densities of CD163^+^ immune cells associate with poor OS, breast cancer specific survival (BCSS), and/or recurrence-free survival (RFS) in patients with primary breast cancer [7–11, 13]. However, there have been contradictory reports including studies indicating no associations between high infiltration of CD163^+^ immune cells and overall and disease-free survival, as well as other studies showing association with improved OS in patients with estrogen receptor (ER)-negative or triple negative (TNBC) tumors [12, 14]. Furthermore, the localization of CD163^+^ immune cells has been shown to be of prognostic importance. Some studies indicate that high densities of CD163^+^ cells in the tumor stroma but not in the tumor nest is associated with poor OS, PFS and/or BCSS [6, 8], while other studies indicate that high CD163^+^ density in the tumor nest associate with unfavorable OS [11]. The presence of CD163^+^ immune cells in different regions may consequently confer different prognostic values, however, there is a lack of studies in metastatic tissue.

Although current literature supports the prognostic potential of CD163^+^ immune cells and associations to adverse clinicopathological features in primary breast cancer, the role of CD163^+^ immune cells in MBC is still unclear. How CD163 expression changes during tumor progression, the prognostic value and potential relevance of the tumoral localization of CD163^+^ immune cells in metastatic tissue remain uncharacterized. In this exploratory study we evaluate CD163^+^ immune cell levels in primary tumors (PT) and corresponding lymph node metastases (LNM) and distant metastases (DM) by immunohistochemistry (IHC) and gene expression (GEX) analyses in a cohort of 139 patients with newly diagnosed MBC. We aim to determine changes and distribution of CD163^+^ immune cell levels in specific tumor regions (tumor nest and tumor stroma) of PT, LNM and DM, and evaluate potential associations of CD163 levels at these distinct sites with clinicopathological characteristics and survival (PFS and OS).

## Materials and methods

### Patient cohort

156 patients with newly diagnosed MBC were included in the prospective observational CTC-MBC trial (clinicalTrials.gov; NCT01322893) conducted in southern Sweden, during 2011–2016, previously described in detail [15]. In short, inclusion criteria were newly diagnosed MBC, age > 18 years, performance status according to Eastern Cooperative Oncology Group (ECOG) 0–2, and a predicted life expectancy of > 2 months. Ethical approval was obtained from the Regional Ethics Committee at Lund University (Dnr 2010/135), and all patients signed a written informed consent. Systemic therapy was decided by the treating physician in accordance with clinical guidelines and patients evaluated approximately every three months, according to clinical routine, for progression versus non-progression using modified Response Evaluation Criteria In Solid Tumors (RECIST) [15, 16]. All clinicopathological data were prospectively collected in case report forms and reported in detail in Larsson et al. [15]. The study was performed in accordance with the REMARK criteria [17].

### Tissue microarray (TMA) and immunohistochemistry (IHC)

**Fig. 1:**
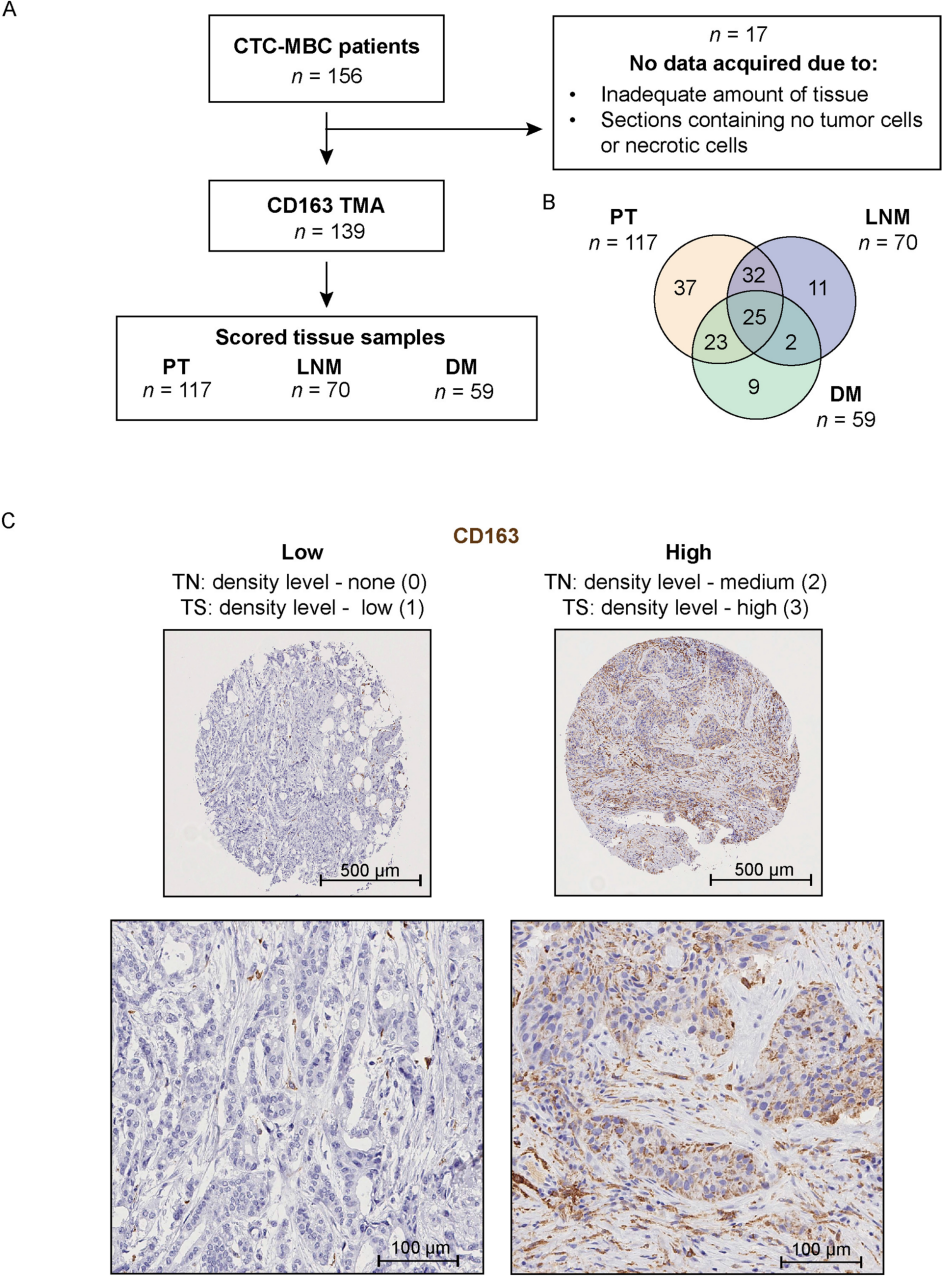
Flowchart of study cohort and representative IHC staining. **A.** Flow chart of patient inclusion for the present study. **B.** Venn diagram of the distribution of patient-matched samples. **C.** Representative IHC staining of CD163 from primary tumors. Left, “low CD163”; none (0) CD163 in the tumor nest and low levels (1) in the tumor stroma. Right, “high CD163”; medium levels (2) of CD163 in the tumor nest and high levels (3) in the tumor stroma. For the majority of analyses, the CD163 levels were dichotomized into low (0/none + low expression (1); *left panel*) and high (medium (2) + high expression (3); *right panel*) in the tumor nest and tumor stroma

Formalin-fixed, paraffin-embedded tissue from primary tumors (PT), lymph node metastases (LNM) and distant metastases (DM) was retrospectively obtained and core biopsies from areas with invasive cancer were constructed into a TMA. Antigen retrieval was performed in a pressure cooker using citrate buffer (pH 6, DAKO) and staining was performed using a validated CD163 antibody (Novocastra, clone 10D, 1:100). The antibody was validated in tonsil using macrophages as positive control and lymphocytes and epithelial cells as negative controls. Tissue samples from 139 patients out of 156 patients recruited were scored for CD163 expression due to inadequate amount of tissue for staining or sections containing no tumor cells or mostly necrotic cells (Fig. 1A, B). Density levels of infiltrating CD163^+^ immune cells in the tumor nest and tumor stroma were semi-quantitatively scored according to four categories (none [0], low [1], medium [2], or high [3] density levels) by two individual observers (KL, FBG) in a blinded manner. Discordant cases were reevaluated and discussed until consensus was reached. Representative IHC stainings of the different density level categories (0–3) are shown in Fig. 1C.

### Gene expression (GEX) analysis of CD163

Tumor tissue from PT, LNM and DM were processed; RNA was isolated and analyzed using the NanoString Breast Cancer 360™ assay on a NanoString nCounter® SPRINT Profiler (NanoString Technologies Inc), as previously described [18, 19]. GEX data generated from the original cohort was primarily used for PAM50 classification [18] and for evaluation of changes in GEX patterns during tumor progression [19]. For the current study, CD163 expression was normalized using the mean and standard deviation of all the samples irrespective of tumor site to enable comparisons across sites. Correlations between CD163 expression and survival in an independent dataset (GEO accession number GSE202203), comprising 3207 patients with primary breast cancer [20], was performed using the publicly available database R2: microarray analysis and visualization platform (http://r2.amc.nl).

### Statistical analyses

Statistical analyses were performed using IBM SPSS Statistics 28 or 29 (correlations to clinicopathological parameters and outcome), and R version 4.3.2 using RStudio (Sankey diagrams and GEX analyses). Sankey plots were constructed using the R package *ggsankey*. Associations between dichotomized CD163^+^ immune cell density levels (“low”: none [0] and low [1], “high”: medium [2] and high [3] levels) and clinicopathological variables were analyzed using Pearson’s chi-squared test or Fisher’s exact test. The relationship between CD163 GEX and IHC was tested using linear regression models. Comparisons of PFS and OS were analyzed using Log-rank test and visualized using Kaplan–Meier curves. Univariate and multivariate Cox regression analyses were used to adjust for established prognostic factors (age at MBC diagnosis, ECOG performance status, histological grade (NHG), subtype, metastasis-free interval (MFI), number of metastatic sites, site of metastasis (non-visceral or visceral) and circulating tumor cells (CTCs) at baseline), as depicted previously [15]. *P*-values < 0.05 were considered statistically significant. Time to progression (PFS) or death of any cause (OS) was calculated from baseline (at study inclusion) and patients without events were censored at last follow-up. Comparison of survival times from primary breast cancer diagnosis to death were calculated from date of initial breast cancer diagnosis and patients without events were censored at last follow-up. Due to the exploratory nature of this study, no analyses to adjust for multiple testing were performed.

## Results

### Patient and tumor characteristics

Of the 156 patients in the original cohort [15], tissue samples from 139 patients with newly diagnosed MBC were scored for density levels of CD163^+^ immune cells (Fig. 1A–C). Patient and tumor characteristics of the CD163 cohort, compared to the original cohort, are summarized in Table 1. The median age at MBC diagnosis was 65 years (range 40–90 years). 94 patients (70.1%) had ER-positive (HER2-negative) disease, 16 (11.9%) had HER2-positive disease and 24 (17.9%) had TNBC as determined by ER and HER2 staining in metastases first-hand and primary tumors (PT) secondly. With regards to PAM50 subtype of the PT, 47 patients (38.5%) had Luminal A, 45 patients (36.9%) Luminal B, 14 patients (11.5%) had HER2-positive, and 16 patients (13.1%) had basal-like primary tumors. 85 patients (61.2%) had an MFI of > 3 years, 26 patients (18.7%) ≤ 3 years, and 28 patients (20.1%) had de novo MBC (*i.e.,* distant metastasis at initial diagnosis). 93 patients (66.9%) had < 3 metastatic sites, 46 patients (33.1%) had ≥ 3 metastatic sites and 82 patients (59.0%) had visceral metastases (metastases in ascites, pleura, liver, lungs or the central nervous system). The median follow-up time was 90 months (range 74–131 months).

**Table 1:** Patient and tumor characteristics of the CD163 cohort compared to the original cohort

Variable	CD163 cohort	Whole original cohort
	Total *n* = 139	Total *n* = 156
Age at MBC diagnosis (years)		
< 65	69 (49.6%)	75 (48.1%)
≥ 65	70 (50.4%)	81 (51.9%)
ECOG at MBC diagnosis		
0	80 (58.8%)	91 (60.7%)
1	35 (25.7%)	37 (24.7%)
2	21 (15.4%)	22 (14.7%)
Unknown	3	6
PT NHG		
I	11 (10.0%)	13 (10.5%)
II	57 (51.8%)	65 (52.4%)
III	42 (38.2%)	46 (37.1%)
Unknown	29	32
PT tumor size		
T1	46 (35.1%)	57 (38.8%)
T2	47 (35.9%)	51 (34.7%)
T3	20 (15.3%)	20 (13.6%)
T4	18 (13.7%)	19 (12.9%)
Unknown	8	9
PT node status		
Negative	35 (28.9%)	44 (32.4%)
Positive	86 (71.1%)	92 (67.6%)
Unknown	18	20
PT subtype (PAM50)		
Luminal A	47 (38.5%)	47 (38.2%)
Luminal B	45 (36.9%)	45 (36.9%)
HER2-pos	14 (11.5%)	15 (12.3%)
Basal	16 (13.1%)	16 (13.1%)
Unknown	17	33
Subtype (IHC) metastases first		
Estrogen receptor-positive (ER^+^, HER2^−^)	94 (70.1%)	105 (69.5%)
HER2-positive	16 (11.9%)	20 (13.2%)
TNBC	24 (17.9%)	26 (17.2%)
Unknown	5	5
Metastasis free interval (MFI)		
0 years (de novo MBC)	28 (20.1%)	31 (19.9%)
> 0 but ≤ 3 years	26 (18.7%)	28 (17.9%)
> 3 years	85 (61.2%)	97 (62.2%)
Metastatic sites, *n*		
< 3	93 (66.9%)	109 (69.9%)
≥ 3	46 (33.1%)	47 (30.1%)
Visceral metastases		
No	57 (41.0%)	65 (41.7%)
Yes	82 (59.0%)	91 (58.3%)
CTC at BL		
< 5	64 (46.0%)	73 (48.0%)
≥ 5	74 (54.0%)	79 (52.0%)
Unknown	1	4

### Distribution and changes in CD163^+^ immune cells during tumor progression

**Fig. 2:**
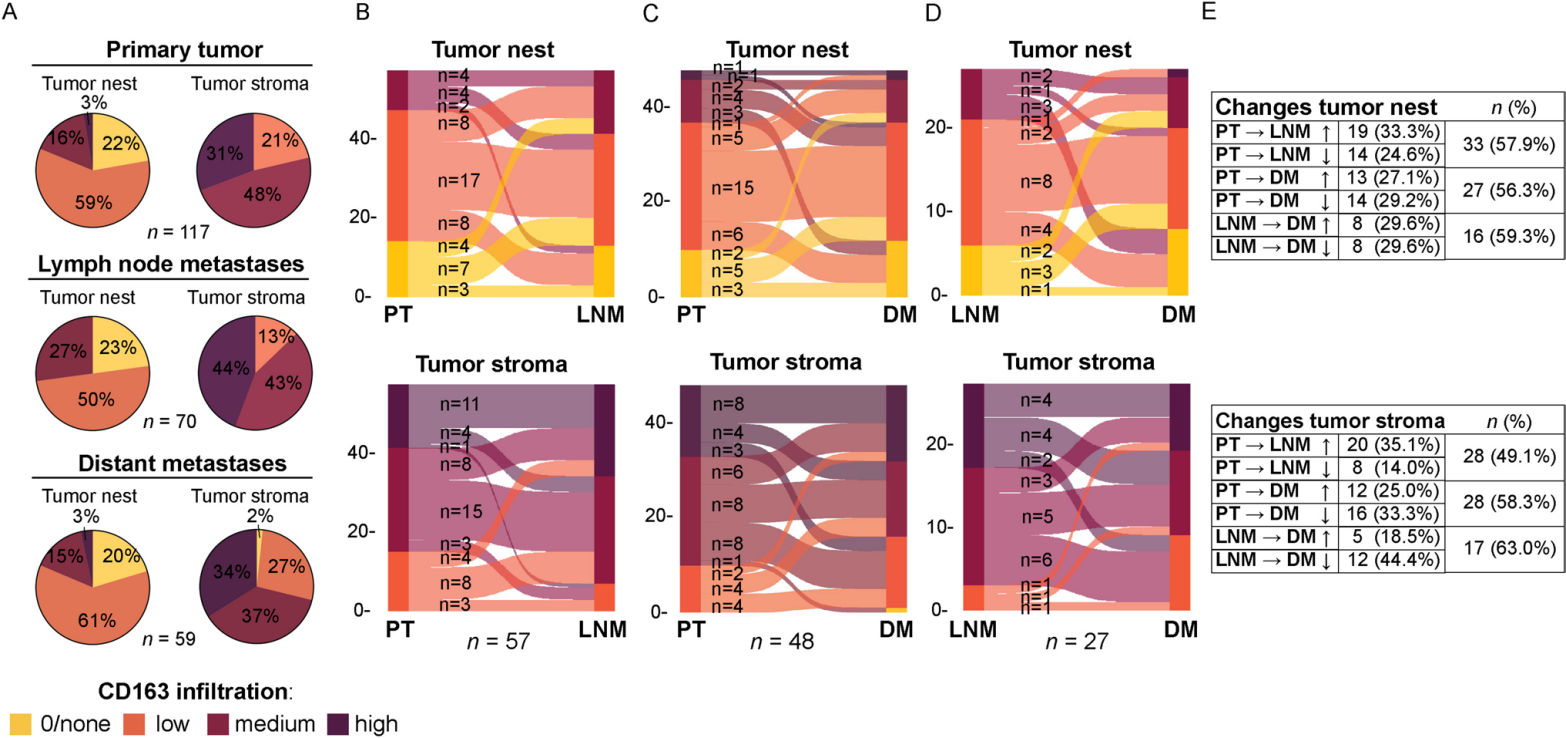
Changes in the levels of CD163^+^ immune cells from primary tumors to lymph node metastases and distant metastases. **A** Pie charts depicting the distribution of CD163 levels and frequency (%) in the tumor nest (*left panels*) and tumor stroma (*right panels*) of primary tumors (PT; *upper panels*), lymph node metastases (LNM; *middle panels*) and distant metastases (DM; *lower panels*). **B**–**D**. Sankey diagram depicting changes in the levels of CD163^+^ cells in the tumor nest (*upper panel*) and tumor stroma (*lower panel*) over time from PT to LNM (B; *n* = 57), PT to DM (C; *n* = 48) and LNM to DM (D; *n* = 27). **E**. Summary of number of patients (*n*) and percentage (%) that display changes in the levels of CD163^+^ immune cells between tumor tissues in the tumor nest (*upper panel*) and tumor stroma (*lower panel*)

The levels of CD163^+^ immune cells were determined in 117 PT, 70 lymph node metastases (LNM) and 59 distant metastases (DM; Fig. 1B). Representative IHC stainings are presented in Fig. 1C. Overall, the density levels of CD163^+^ immune cells were higher in the tumor stroma compared to the tumor nest, but the patterns were similar across the tumor locations (PT, LNM and DM; Fig. 2). Next, we visualized changes in CD163^+^ immune cells in the tumor nest (Fig. 2B–E, upper panels) and tumor stroma (Fig. 2B–E, lower panels) during tumor progression using Sankey diagrams of patient-matched tissue samples. Approximately half of the patients displayed changes in the density levels of CD163^+^ immune cells between the sites (Fig. 2B–E), however, these changes were not significant as determined by McNemar analysis (data not shown). To summarize, distribution of CD163^+^ immune cells is similar across tumor sites (PT, LNM and DM) and observed changes during tumor progression were not significant.

### CD163^+^ immune cells in the primary tumor associate to clinical parameters and gene expression (GEX)

Next, we dichotomized the levels of CD163^+^ immune cells into “low” (none [0] + low [1] density level) and “high” (medium [2] + high [3] density level; Fig. 1C) and analyzed possible associations to clinicopathological variables (Table 2). High levels of CD163^+^ immune cells in the PT nest associated significantly with higher NHG (*P* = 0.023), intrinsic PAM50 basal-like subtype (*P* = 0.007) and a shorter MFI (*P* = 0.040; Table 2, left panel). Similarly, high levels of CD163^+^ immune cells in the PT stroma associated with higher tumor grade (*P* = 0.030) and molecular subtype Luminal B and basal-like. In contrast, low levels of CD163^+^ immune cells associated with Luminal A subtype (*P* = 0.003) (Table 2, right panel). With regards to the levels of CD163^+^ immune cells in the tumor nest and stroma of LNM and DM, only a few significant associations were observed (data not shown); low levels of CD163^+^ immune cells in the LNM stroma associated with presence of visceral metastases (*P* = 0.009) whereas high levels of CD163^+^ immune cells in the DM tumor stroma associated with more metastatic sites (≥ 3 metastatic sites *P* = 0.048).

**Table 2:** Correlations between CD163^+^ immune cells in the primary tumor nest (*left*) and stroma (*right*) and clinical parameters

Variable	Total *n* = 117	Primary tumor (PT) nest			Primary tumor (PT) stroma		
		Low CD163 *n* = 95	High CD163 *n* = 22	*P*-value	Low CD163 *n* = 25	High CD163 *n* = 92	*P*-value
Age (years)							
< 65	57	45 (47.4%)	12 (54.5%)	0.544^a^	12 (48%)	45 (48.9%)	0.935^a^
≥ 65	60	50 (52.6%)	10 (45.5%)		13 (52%)	47 (51.1%)	
Baseline ECOG							
0	68	59 (62.8%)	9 (40.9%)	0.129^b^	19 (76.0%)	49 (53.8%)	0.154^b^
1	30	21 (22.3%)	9 (40.9%)		4 (16.0%)	26 (28.6%)	
2	18	14 (14.9%)	4 (18.2%)		2 (8.0%)	16 (17.6%)	
Unknown	1	1	0		0	1	
PT NHG							
I	8	8 (10.8%)	0 (0.0%)	**0.023**^b^	2 (10.5%)	6 (8.1%)	**0.030**^b^
II	47	41 (55.4%)	6 (31.6%)		14 (73.7%)	33 (44.6%)	
III	38	25 (33.8%)	13 (68.4%)		3 (15.8%)	35 (47.3%)	
Unknown	24	21	3		6	12	
PT tumor size							
T1	39	32 (35.6%)	7 (31.8%)	0.894^b^	9 (37.5%)	30 (34.1%)	0.722^b^
T2	41	33 (36.7%)	8 (36.4%)		8 (33.3%)	33 (37.5%)	
T3	17	14 (15.6%)	3 (13.6%)		5 (20.8%)	12 (13.6%)	
T4	15	11 (12.2%)	4 (18.2%)		2 (8.3%)	13 (14.8%)	
Unknown	5	5	0		1	4	
PT node status							
Negative	32	23 (28.0%)	9 (42.9%)	0.191^a^	6 (27.3%)	26 (32.1%)	0.664^a^
Positive	71	59 (72.0%)	12 (57.1%)		16 (72.7%)	55 (67.9%)	
Unknown	14	13	1		3	11	
PT subtype (PAM50)							
Luminal A	45	41 (43.6%)	4 (18.2%)	**0.007**^b^	17 (70.8%)	28 (30.4%)	**0.003**^b^
Luminal B	43	35 (37.2%)	8 (36.4%)		3 (12.5%)	40 (43.5%)	
HER2-positive	12	10 (10.6%)	2 (9.1%)		2 (8.3%)	10 (10.9%)	
Basal	16	8 (8.5%)	8 (36.4%)		2 (8.3%)	14 (15.2%)	
Unknown	1	1	0		1	0	
BC subtype (IHC) based on metastasis first							
ER-positive	81	66 (73.3%)	15 (68.2%)	0.052^b^	20 (83.3%)	61 (69.3%)	0.388^b^
HER2-positive	11	11 (12.2%)	0 (0.0%)		2 (8.3%)	9 (10.2%)	
TNBC	20	13 (14.4%)	7 (31.8%)		2 (8.3%)	18 (20.5%)	
Unknown	0	0	0		1	4	
Time from PT to metastasis (MFI)							
0 (de novo MBC)	23	20 (21.1%)	3 (13.6%)	**0.040**^b^	3 (12.0%)	20 (21.7%)	0.300^b^
> 0 but ≤ 3	23	14 (14.7%)	9 (40.9%)		3 (12.0%)	20 (21.7%)	
> 3	71	61 (64.2%)	10 (45.5%)		19 (76.0%)	52 (56.5%)	
Metastatic sites, *n*							
< 3	78	65 (68.4%)	13 (59.1%)	0.403^a^	18 (72.0%)	60 (65.2%)	0.524^a^
≥ 3	39	30 (31.6%)	9 (40.9%)		7 (28.0%)	32 (34.8%)	
Metastatic localization							
Visceral	49	40 (42.1%)	9 (40.9%)	0.918^a^	12 (48.0%)	37 (40.2%)	0.484^a^
Non-visceral Visceral	68	55 (57.9%)	13 (59.1%)		13 (52.0%)	55 (59.8%)	
CTC at baseline							
< 5	55	47 (49.5%)	8 (36.4%)	0.267^a^	9 (36.0%)	46 (50.0%)	0.214^a^
≥ 5	62	48 (50.5%)	14 (63.6%)		16 (64.0%)	46 (50.0%)	

High and low levels of CD163^+^ immune cells in the primary tumor nest and stroma were associated with clinical parameters. Statistics by a. Pearson Chi-squared or b. Fisher’s exact test when expected counts were < 5 in ≥ 1 cell. *P*-values < 0.05 are highlighted in bold

In conjunction with previous GEX analyses of the original cohort for PAM50 classification [18] and evaluation of changes in GEX patterns during tumor progression [19], we also obtained information regarding CD163 GEX on the same matched material. This allowed us to analyze the concordance between CD163 levels as assessed by IHC and GEX. Overall, CD163 GEX levels from bulk tissue correlated well with the four scoring categories of CD163 by IHC (density level none [0], low [1], medium [2] and high [3]) in tumor stroma at all sites (PT, LNM and DM; Supplemental Fig. 1A–C). These results indicate that high density levels of CD163^+^ immune cells in the tumor nest and stroma of the PT, and less so in the DM, associate with adverse clinical features. Furthermore, there is a high degree of concordance between CD163 levels as assessed by IHC and GEX.

### High levels of CD163^+^ immune cells in the primary tumor, but not in metastases, associate with worse outcome

**Fig. 3:**
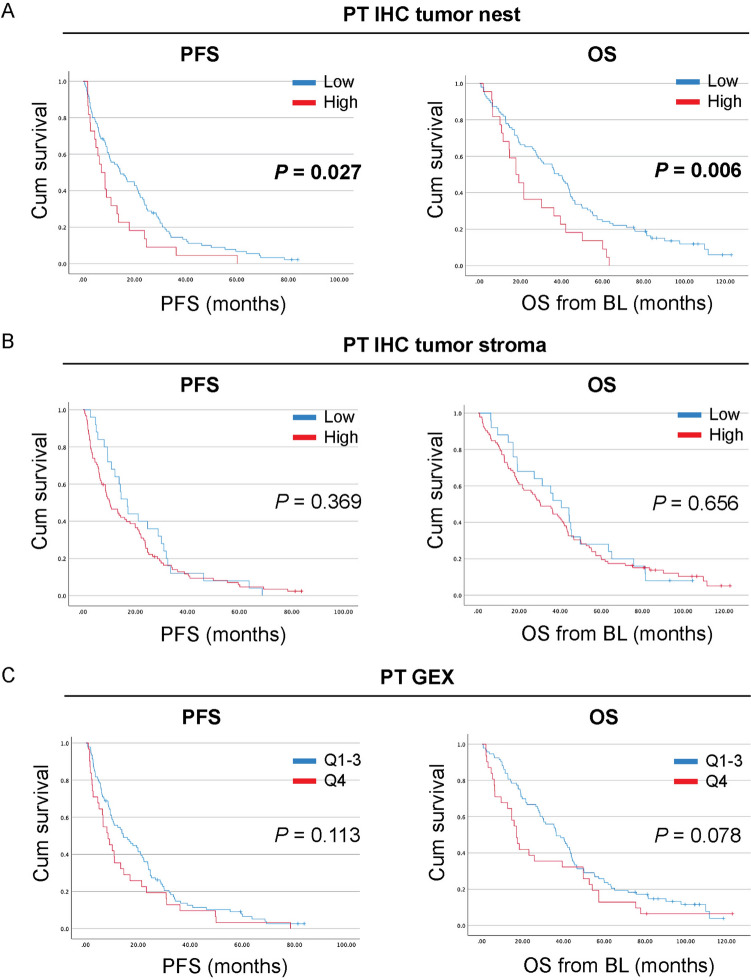
High levels of CD163^+^ immune cells in the tumor nest of primary tumors associate with shorter survival. Kaplan–Meier curves with log-rank test of progression-free survival (PFS; *left* panels) and overall survival (OS; *right panels*) from baseline according to the levels of CD163^+^ cells in **A,** the primary tumor (PT) nest, **B**, PT stroma, or **C**, according to CD163 gene expression (GEX). *P-*values < 0.05 are highlighted in bold

To further determine potential prognostic relevance of CD163^+^ immune cells in PT, LNM and DM of patients with MBC, we next performed log-rank tests illustrated with Kaplan–Meier curves to compare PFS and OS in patients with high or low levels (dichotomized) of CD163^+^ immune cells as determined by IHC or GEX (Fig. 3, and Supplemental Fig. 2). High levels of CD163^+^ immune cells in the PT nest associated significantly with shorter PFS (*P* = 0.027; 7.1 months 3.9–10.4 95% CI versus 14.6 months 8.5–20.6 95% CI; Fig. 3A, left panel) as well as with shorter OS (*P* = 0.006; 17.8 months 11.4–24.1 95% CI versus 38.6 months 28.5–48.8 95% CI; Fig. 3A, right panel). No significant associations were observed for CD163^+^ immune cells in the PT stroma (Fig. 3B). When analyzing the prognostic potential of CD163 GEX in PT from patients with MBC, high levels of CD163 GEX (expression dichotomized based on quartiles 1–3 vs. quartile 4) in the PT tended to associate with worse PFS (*P* = 0.113) and OS (*P* = 0.078), however these results were not statistically significant (Fig. 3C).

When analyzing PFS and OS in relation to CD163 levels in LNM and DM by IHC and GEX, no significant associations were observed (Supplemental Fig. 2). Combined, these results indicate that CD163^+^ immune cells in the tumor nest of PT associate with worse outcome (PFS and OS) in MBC, while the prognostic relevance of CD163 at other tumor sites and in the PT stroma was not significant.

### CD163^+^ immune cells in primary tumors associate with survival in ER-positive MBC

**Fig. 4:**
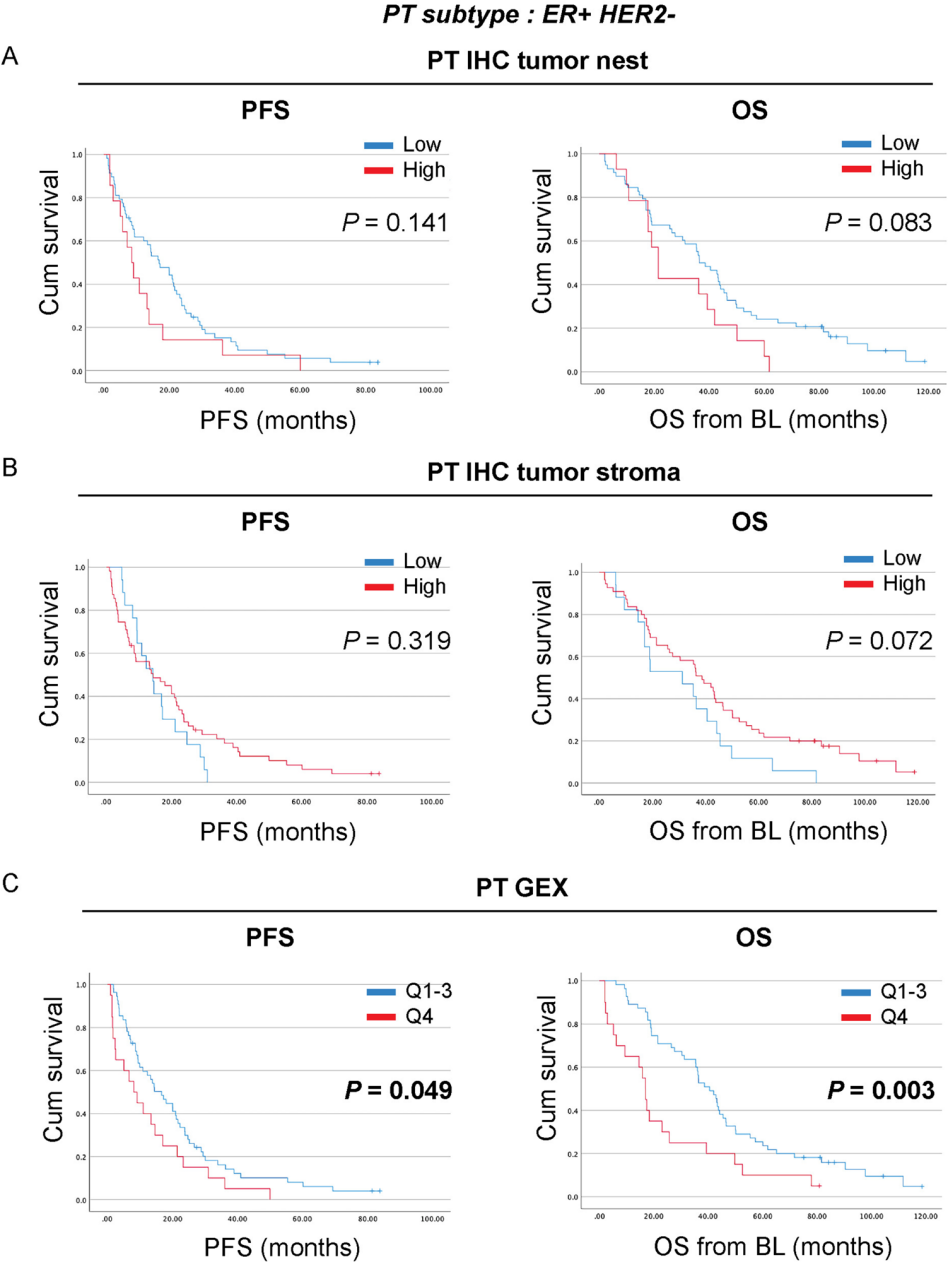
High levels of CD163 GEX in the primary tumor associate with shorter survival in patients with ER^+^HER2^−^ primary tumors. Patients were stratified according to ER^+^HER2^−^ subtype of the primary tumor (PT). Kaplan–Meier curves with log-rank test of progression-free survival (PFS; *left panels*) or overall survival (OS; *right panels*) according to the levels of CD163^+^ immune cells in the **A**, PT tumor nest, **B**, PT tumor stroma, or **C**, CD163 GEX of PT. *P-*values < 0.05 are highlighted in bold

As the breast cancer subtype also has been proposed to associate with CD163 [7–9, 11, 12], we next investigated the prognostic impact of CD163^+^ immune cells in the PT, stratified according to IHC-subtype as this was used to determine MBC treatment (Fig. 4). For ER-positive MBC, high density levels of CD163^+^ immune cells in the tumor nest and stroma did not significantly correlate to PFS or OS (Fig. 4A, B). However, high CD163 GEX in ER-positive PT was significantly associated with shorter PFS and OS (*P* = 0.049, estimated median PFS for patients with high and low levels of CD163-GEX; 8.0 months 2.7–13.4 95% CI versus 16.6 months 11.0–22.3 95% CI, respectively and *P* = 0.003 estimated median OS 17.0 months 14.4–19.5 95% CI versus 40.5 months 33.3–47.8 95% CI, respectively; Fig. 4C). As for HER2^+^ and triple-negative (ER^−^HER2^−^, TNBC) subtypes, these subgroups were small with few events and therefore survival analyses were inconclusive (data not shown). Similarly, when analyzing the levels of CD163^+^ immune cells in LNM and DM stratified according to IHC-subtype, no conclusive results were observed due to small subgroups with few events (data not shown). Altogether, these results indicate that high levels of CD163 GEX in the PT associate with worse prognosis, primarily in ER-positive subtype.

### Uni- and multivariable Cox regression analyses

Next, we performed uni- and multivariable Cox regression analyses to determine the potential of infiltrating CD163^+^ immune cells to act as an independent prognostic factor in MBC (Table 3). Univariable Cox regression analyses indicate that high levels of CD163^+^ immune cells in the PT nest associated with shorter PFS and OS (*P* = 0.029, HR_PFS_: 1.692 95% CI: 1.056–2.712 and *P* = 0.007, HR_OS_: 1.943 95% CI: 1.200–3.144, respectively; Table 3). However, after adjusting for established prognostic factors in multivariable analyses, these associations were not significant. Associations between CD163^+^ immune cells in the tumor nest of LNM and PFS and OS were significant in multivariable analyses (Table 3) (*P* = 0.006, HR_PFS-adj_: 0.361 95% CI: 0.176–0.743 and *P* = 0.003, HR_OS-adj_: 0.286 95% CI: 0.127–0.645) as well as CD163 GEX of DM and PFS and OS (*P* = 0.019, HR_PFS-adj_: 2.551 95% CI: 1.164–5.589 and *P* = 0.036, HR_OS-adj_: 2.424 95% CI: 1.061–5.537). This should, however, be interpreted with care as these associations were not significant in univariable analyses. Due to the exploratory nature of the study, no testing for multiple comparisons (FDR or Bonferroni) were made. Altogether, these data indicate that the levels of CD163^+^ immune cells in the PT may identify MBC patients with worse outcome. However, these observations were not independent of other prognostic factors and the results need to be validated in a larger cohort.

**Table 3:** Cox regression hazard ratios for the levels of CD163^+^ immune cells in the tumor nest and tumor stroma and CD163 GEX in relation to progression-free and overall survival

Variable	Progression-free survival				Overall survival			
	Hazard ratio (95% CI)				Hazard ratio (95% CI)			
	Unadjusted	*P*-value	Adjusted	*P*-value	Unadjusted	*P*-value	Adjusted	*P*-value
Primary tumor								
CD163 in tumor nest	1.692 (1.056–2.712)	**0.029**	1.558 (0.829–2.930)	0.169	1.943 (1.200–3.144)	**0.007**	1.219 (0.637–2.332)	0.55
CD163 in tumor stroma	1.226 (7.85–1.916)	0.371	1.220 (0.679–2.190)	0.506	1.111 (0.699–1.766)	0.657	1.014 (0.545–1.887)	0.964
CD163 GEX	1.391 (0.923–2.098)	0.115	1.528 (0.855–2.730)	0.153	1.462 (0.956–2.237)	0.08	1.767 (0.986–3.168)	0.056
Lymph node metastases								
CD163 in tumor nest	0.606 (0.348–1.057)	0.078	0.361 (0.176–0.743)	**0.006**	0.672 (0.380–1.189)	0.172	0.286 (0.127–0.645)	**0.003**
CD163 in tumor stroma	1.170 (0.576–2.376)	0.663	1.217 (0.433–3.421)	0.709	0.794 (0.390–1.617)	0.525	0.834 (0.307–2.268)	0.723
CD163 GEX	1.139 (0.649–2.000)	0.65	1.065 (0.417–2.724)	0.895	1.191 (0.676–2.099)	0.545	1.217 (0.407–3.639)	0.725
Distant metastases								
CD163 in tumor nest	1.020 (0.526–1.976)	0.953	0.661 (0.240–1.824)	0.425	0.991 (0.509–1.929)	0.979	0.658 (0.223–1.942)	0.449
CD163 in tumor stroma	1.580 (0.872–2.861)	0.132	1.158 (0.496–2.704)	0.735	1.637 (0.874–3.069)	0.124	1.105 (0.451–2.707)	0.828
CD163 GEX	1.210 (0.715–2.049)	0.478	2.551 (1.164–5.589)	**0.019**	1.145 (0.668–1.963)	0.623	2.424 (1.061–5.537)	**0.036**

Dichotomized levels of CD163^+^ immune cells in the tumor nest, in the tumor stroma, or of CD163 gene expression (GEX) associations with progression-free (*left*) or overall survival (*right*) in univariable (unadjusted) or multivariable analyses (adjusted for age at MBC diagnosis, ECOG performance status, NHG, breast cancer subtype based on metastases firsthand, MFI, number of metastatic sites (< 5 or ≥ 3 sites), presence of visceral metastases and the number of CTCs at MBC diagnosis (< 5 or ≥ 5 CTCs)). CI = confidence interval. *P*-values < 0.05 are highlighted in bold

### High levels of CD163^+^ immune cells in the primary tumor associate with shorter survival from the initial breast cancer diagnosis

**Fig. 5:**
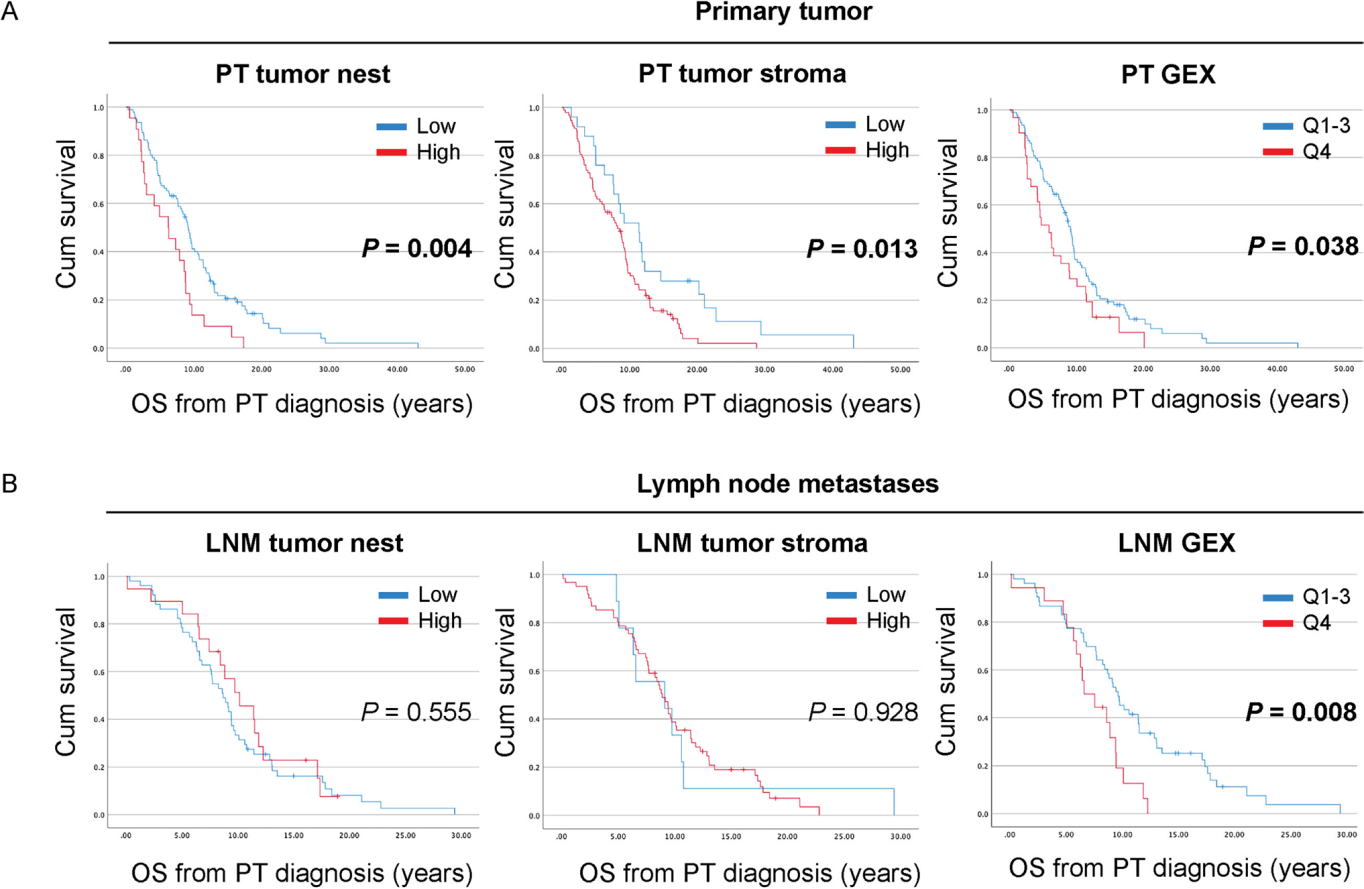
High levels of CD163^+^ immune cells in the primary tumor associate with shorter survival from primary tumor diagnosis. Kaplan–Meier curves with log-rank test of overall survival (OS) from initial primary tumor (PT) diagnosis according to infiltration of CD163^+^ cells in the tumor nest, tumor stroma, or CD163 gene expression (GEX) of PT (**A**) or synchronous lymph node metastases (LNM; **B**). *P-*values < 0.05 are highlighted in bold

Finally, in order to investigate the potential of CD163 to predict survival from the initial breast cancer diagnosis (PT diagnosis) we next compared survival (MFI plus OS from time of MBC diagnosis) in patients with high or low levels of CD163^+^ immune cells in the tumor nest, tumor stroma, or of CD163 GEX in the PT (Fig. 5A) or synchronous LNM (Fig. 5B). In accordance with previous results, high levels of CD163^+^ immune cells in the PT nest (*P* = 0.004, estimated median OS from PT diagnosis; high levels; 6.3 years, 2.6–9.9 95% CI and low levels; 9.2 years 8.1–10.2 95% CI, respectively), as well as the PT stroma (*P* = 0.013, estimated median OS from PT diagnosis; 8.2 years, 6.8–9.7 95% CI versus 11.4 years 6.8–16.0 95% CI, respectively) were associated with worse OS (from initial diagnosis). Patterns were similar for CD163 GEX (*P* = 0.038; Fig. 5A). No significant difference was observed in patients with high or low levels of CD163^+^ immune cells in LNM (Fig. 5B). However, high levels of CD163 GEX in synchronous LNM associated with shorter OS from the initial breast cancer diagnosis (*P* = 0.008, estimated median OS from PT diagnosis; 6.6 years, 4.3–8.9 95% CI versus 9.6 years 8.3–11.0 95% CI, respectively; Fig. 5B). In accordance with this, using an independent dataset (GEO accession number GSE202203) comprising primary tumor GEX from 3207 patients with early breast cancer [20], high levels of CD163 GEX (quartiles 1–3 *versus* quartile 4) associated with shorter relapse-free survival and overall survival (*P* = 0.009 and *P* = 0.00007, respectively; Supplemental Fig. 3). These results indicate that the density levels of CD163^+^ immune cells as well as CD163 GEX in PT have potential to predict OS already at time of initial breast cancer diagnosis.

## Discussion

In this study, we utilize a unique cohort to investigate how the levels of CD163^+^ immune cells change during tumor progression (from PT to LNM and DM) and determine the clinical relevance of CD163^+^ immune cells with regards to associations with clinicopathological factors and disease outcomes in MBC. To our knowledge, this is the first study to determine the prognostic potential of CD163^+^ immune cells in patients with MBC. Our data indicate that high levels of CD163^+^ immune cells in PT, but not LNM or DM, associate with adverse clinical features (including higher grade, basal-like subtype and shorter MFI) and poor outcome (PFS and OS).

CD163 is a marker for anti-inflammatory myeloid cells, comprising predominantly TAMs but also myeloid-derived suppressor cells (MDSCs) [4, 5]. CD163^+^ immune cells are well-established players in the tumor microenvironment and several studies have reported associations with poor clinical features and outcome in primary breast cancer [7–12]. However, their role in metastatic disease remains unclear. We have previously shown that circulating monocytic MDSCs (Mo-MDSCs), that also may express CD163, are enriched in the peripheral blood of MBC patients and associate with adverse prognostic features [21, 22]. In the present study, we found that the overall distribution of CD163^+^ immune cells was similar between PT, LNM and DM, although statistically insignificant shifts were observed during tumor progression. High levels of CD163^+^ immune cells in the PT, but not LNM or DM, were associated with adverse clinicopathological variables such as higher PT grade (NHG) and basal-like and/or Luminal B subtypes. This is in agreement with previous studies in primary breast cancers reporting associations to higher histological grade and hormone-receptor negative subtype [7–12]. It should be noted that the PT samples in our cohort are from patients that all developed metastatic disease, which may explain the discrepancies regarding associations with Luminal B subtype from previous studies focusing only on primary breast cancer.

This study also indicates that high levels of CD163^+^ immune cells in the PT nest associated with shorter MFI as well as PFS and OS in MBC patients. These observations were, however, not significant after adjusting for other established prognostic factors. In addition, both high protein levels (IHC data) and GEX of CD163 in PT strongly associated with shorter survival from the time of the initial breast cancer diagnosis (PT diagnosis). The association between high levels of CD163 GEX and shorter survival was also validated in an independent dataset of patients with primary, early breast cancer. Interestingly, the levels of CD163^+^ immune cells in PT had a stronger association to prognosis than levels in DM. While the patterns of prognostic potential of CD163 appear to be different in PT and DM, it is important to note that analyses of PT are better powered due to larger sample size.

The observation that CD163^+^ immune cell levels of the PT are of prognostic value also for MBC has several important potential future implications that should be further investigated. CD163 may add prognostic information in MBC, as it identifies a subgroup of patients with worse prognosis that could benefit from more frequent monitoring and potential treatment adjustments. Furthermore, although molecular profiling of distant metastases guides the treatment strategy in MBC today, material from metastases may not always be available. Thus, the possibility to use information regarding CD163^+^ immune cells in PT tissue is of clinical relevance. In line with this, our recent study from the same MBC cohort indicated that the GEX profile of the PT is as useful as the DM for predicting outcome in MBC [19]. In addition, the possibility of targeting CD163^+^ immune cells, potentially already at the time of the PT diagnosis, may pose an interesting and future treatment strategy to prevent MBC and prolong survival. CD163 is upregulated in several conditions, ranging from acute and chronic inflammatory diseases to malignancies [4]. Current efforts in targeting CD163^+^ immune cells focus on anti-CD163 antibodies directly conjugated to cytotoxic drugs or anti-CD163 coated liposomes loaded with drugs. These studies have shown effects in reducing tumor growth and affect the local immune composition, as well as to reduce the metastatic spread in mouse models of cancers such as melanoma and ovarian carcinoma [4]. The findings that CD163 GEX hold prognostic value also in ER-positive MBC is highly relevant since most immunotherapies currently used are primarily aimed at TNBC. To further explore the role of CD163^+^ immune cells in the different molecular subtypes is therefore of importance.

This study makes use of a unique cohort of matched tissue samples (PT, LNM and DM) providing both GEX and IHC analyses, from 139 MBC patients planned for first line systemic therapy with long-term follow-up. One limitation is the cohort size, and the analyses performed should thus be considered exploratory. Our data should be validated in a larger MBC cohort to facilitate subgroup analyses of CD163^+^ immune cells in different molecular subtypes of MBC as well as in metastases from distinct localizations (e.g., in bone or liver metastases). Understanding the immunological heterogeneity in metastases compared to PT will be of great importance for improving current prognostic tools as well as for future individualized therapeutic strategies.

To conclude, high levels of CD163^+^ immune cells as well as CD163 GEX in the PT rather than in metastases associate with adverse clinicopathological factors and shorter PFS and OS in MBC as well as with shorter OS from time of initial PT diagnosis. However, these associations were not significant after multivariable analyses adjusting for other established prognostic factors. These results indicate that CD163^+^ immune cells in the PT may have potential to add prognostic information for clinical outcome in MBC patients, however there is need for validation in larger cohorts.

## Supplementary Information

Below is the link to the electronic supplementary material.

Supplementary file1 (PDF 4042 KB)

Supplementary file2 (PDF 15 KB)

## Data Availability

No datasets were generated or analysed during the current study.
