# Supplementary material for: Prognostic relevance of CD163+ immune cells in patients with metastatic breast cancer
Source: Cancer Immunol Immunother. 2025 Jan 3;74(2):42. doi: 10.1007/s00262-024-03892-2 (PMC11699000; doi:10.1007/s00262-024-03892-2)

Supplemental Figure 1

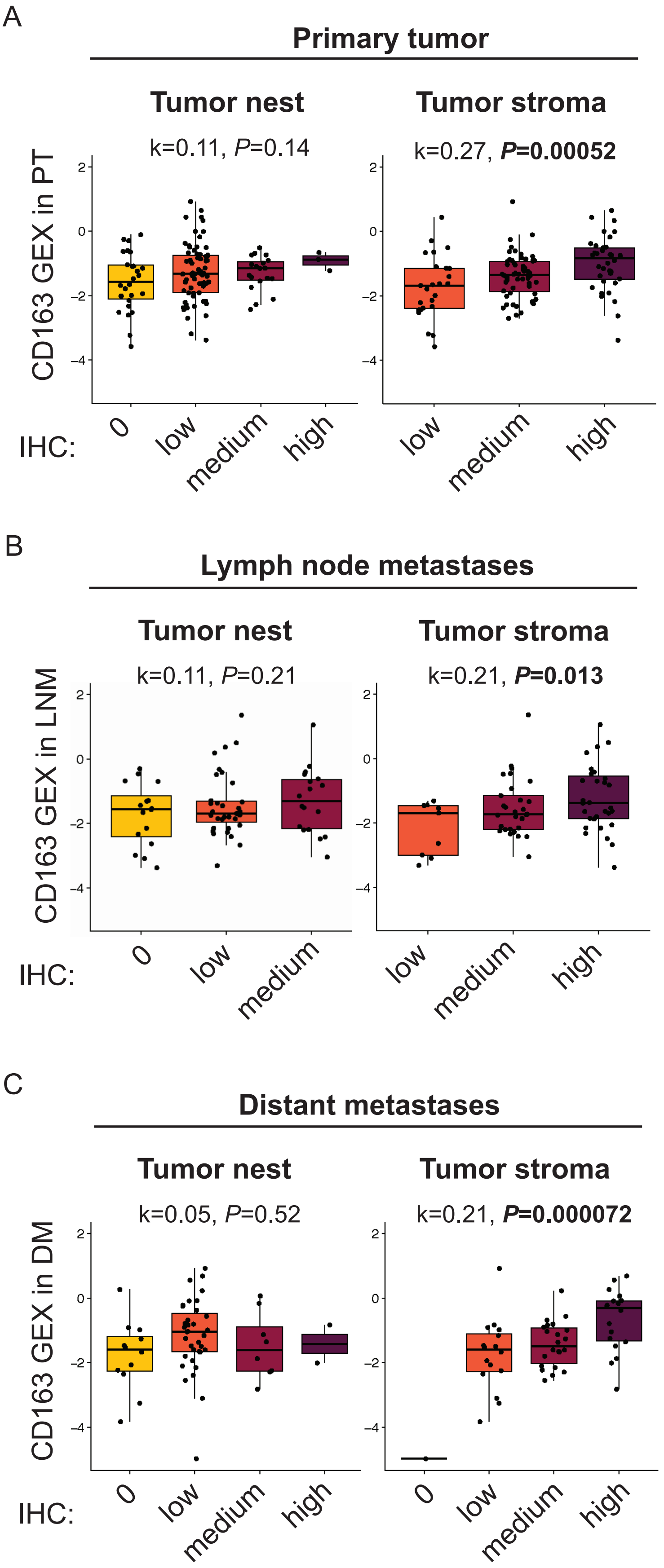

Supplemental Figure 2

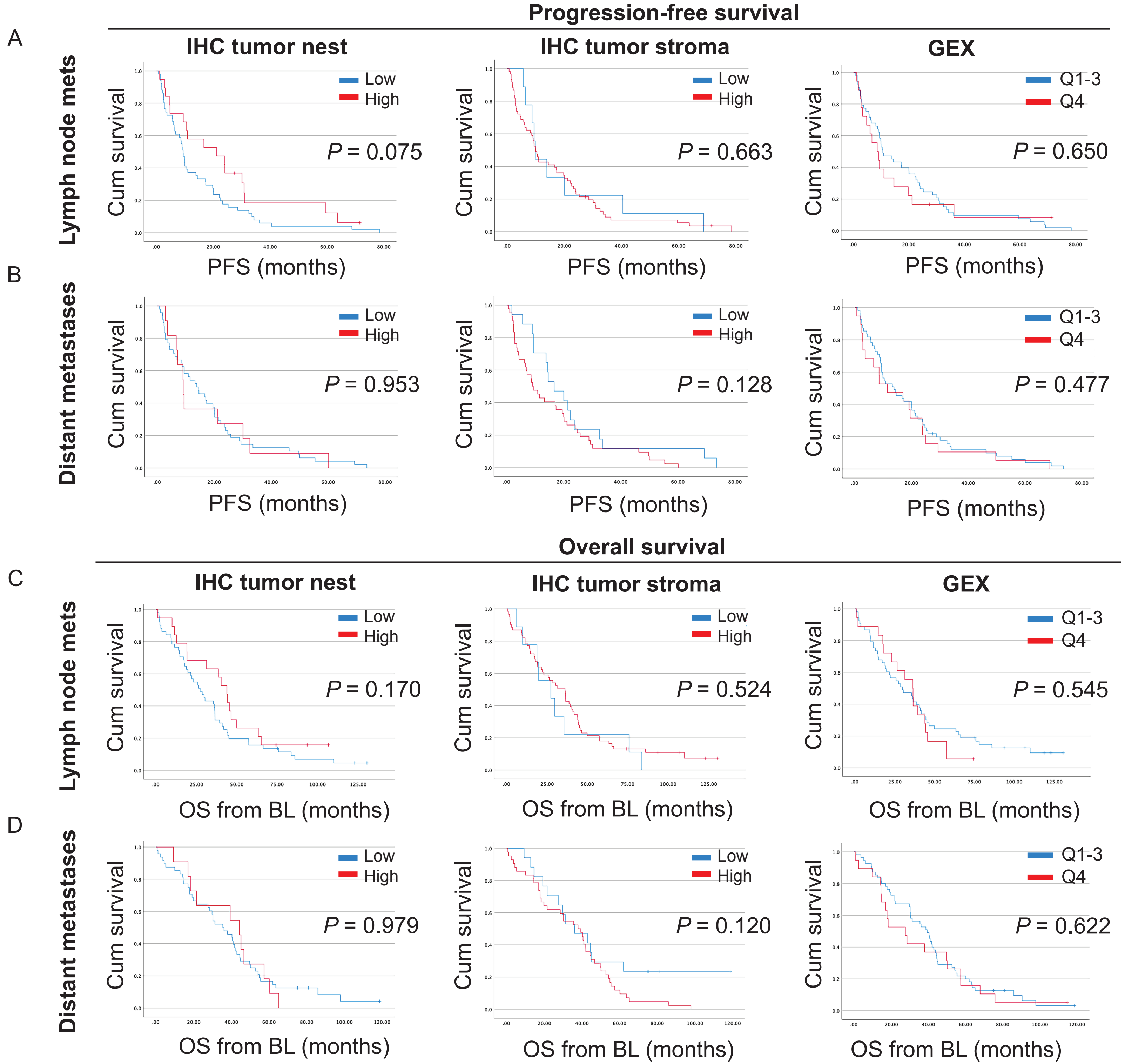

Supplemental Figure 3

**Primary breast cancer : PT GEX**

GEO accession no: GSE202203

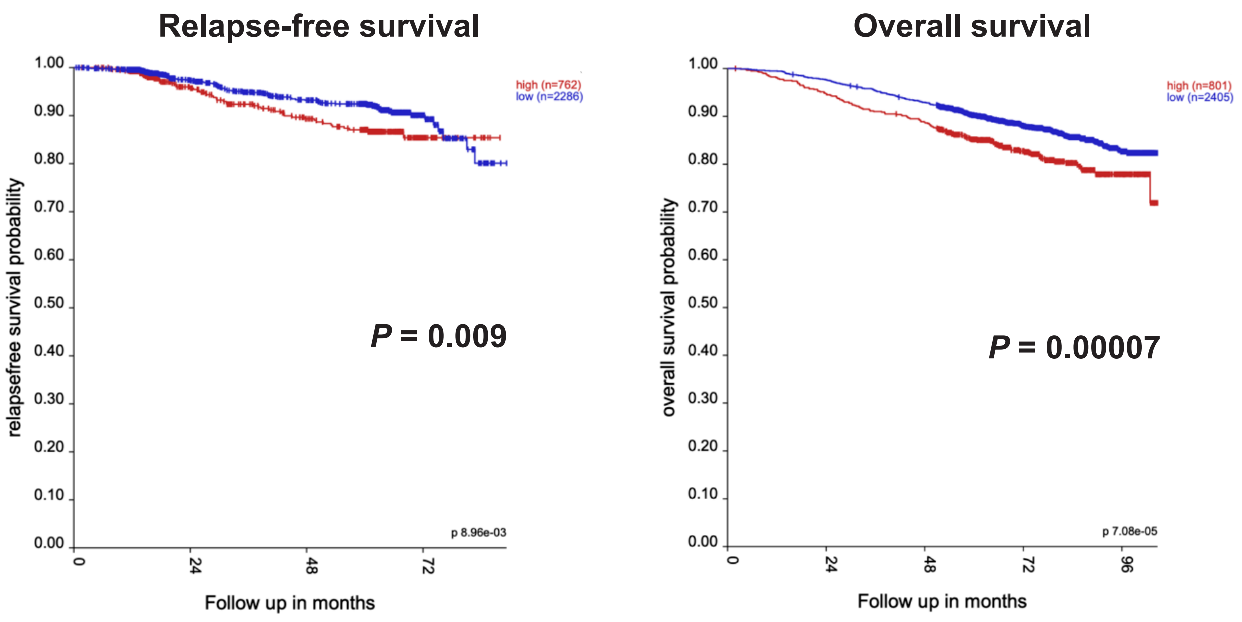

Supplement: Supplementary file 1 — Supplementary file1 (PDF 4042 KB) [file 262_2024_3892_MOESM1_ESM.pdf]
