# Supplementary material for: Prognostic relevance of CD163+ immune cells in patients with metastatic breast cancer
Source: Cancer Immunol Immunother. 2025 Jan 3;74(2):42. doi: 10.1007/s00262-024-03892-2 (PMC11699000; doi:10.1007/s00262-024-03892-2)
Supplement: Supplementary file 2 — Supplementary file2 (PDF 15 KB) [file 262_2024_3892_MOESM2_ESM.docx]

**SUPPLEMENTAL FIGURE LEGENDS**

**Supplemental Figure 1. The levels of CD163 show a high degree of concordance when analyzed by GEX versus IHC.** The relative gene expression (GEX) levels of CD163 according to IHC scoring category (0/none, low, medium and high) in the tumor nest (*left*) and tumor stroma (*right*) of primary tumors (**A**), lymph node metastases (LNM; **B**) and distant metastases (DM; **C**). Statistics by linear regression with estimates (k) and *P*-values indicated.

**Supplemental Figure 2. The levels of CD163^+^ cells in lymph node- or distant metastases do not associate significantly with survival.** Kaplan-Meier curves with log-rank test of progression-free survival (PFS; **A-B**) and overall survival (OS; **C-D**) according to the levels of CD163^+^ cells (IHC) in the tumor nest or tumor stroma, or according to CD163 GEX (stratified by quartiles; quartile 1-3 *versus* quartile 4) in lymph node metastases (LNM; **A, C**) or distant metastases (DM; **B, D**). *P-*values < 0.05 are highlighted in bold.

**Supplemental Figure 3. High CD163 GEX in primary tumors from patients with early breast cancer associate with shorter survival**. Correlation between CD163 GEX and relapse-free survival (*left*) and overall survival (*right*) was determined in an independent dataset (GEO accession number GSE202203) comprising primary tumors (PT) from 3207 patients with primary, early, breast cancer (20) using the publicly available database R2: microarray analysis and visualization platform (http://r2.amc.nl).
